# Transcriptome Profiles of Highly Pathogenic Pure Avian H7N9 Virus-Infected Lungs of BALB/c Mice

**DOI:** 10.3389/fvets.2020.603584

**Published:** 2020-12-21

**Authors:** Wenxiao Gong, Kun Huang, Yufei Zhang, Xinglin He, Chengfei Li, Haiying Mao, Yanming Wei, Zhong Zou, Meilin Jin

**Affiliations:** ^1^State Key Laboratory of Agricultural Microbiology, Huazhong Agricultural University, Wuhan, China; ^2^College of Veterinary Medicine, Huazhong Agricultural University, Wuhan, China; ^3^Key Laboratory of Development of Veterinary Diagnostic Products, Ministry of Agriculture, Wuhan, China

**Keywords:** highly pathogenic H7N9 avian influenza virus, yangtze river delta lineage, over-activated antiviral signals, transcriptome, pathogenicity

## Abstract

Avian influenza A (H7N9) viruses emerged in China in 2013 and caused a zoonotic disease associated with a high case-fatality ratio of more than 30%. Transcriptional profiles obtained using animal models reveal host responses to the disease, thereby providing insights into disease pathogenesis. Therefore, we aimed to characterize the host responses of the H7N9 virus infected-mouse lungs in this study. First, we isolated an avian-originated H7N9 strain, which was shown to be highly pathogenic to both chickens and mice. Genomic analysis results suggested that a 12-nucleotide-insertion was present at the hemagglutinin cleavage site, and both the hemagglutinin and neuraminidase genes belonged to the Yangtze River Delta lineage. RNA sequencing results revealed 566 differentially expressed genes in the H7N9-infected lungs. Moreover, transcriptome analysis revealed that over-activated antiviral signals and intense interferon-stimulated gene products possibly contributed to the high virulence of the virus in mice. Importantly, lung concentrations of inflammatory cytokines, including interleukin-1β and interleukin-6, interferon-β, and tumor necrosis factor-α, were upregulated in response to H7N9 virus infection. Overall, the present study provided a comprehensive understanding of H7N9 virus pathogenicity and correlated host immune responses.

## Introduction

Influenza A virus (IAV) is an enveloped, segmented, negative-strand RNA virus belonging to the Orthomyxoviridae family (1). IAVs have a wide range of hosts, including humans, wild birds, poultry, and pigs; wild waterfowl are generally considered IAV reservoirs (2). The segmented pattern of the IAV genome and the proofreading activity-lacking nature of the viral polymerase allowed the accumulation of nucleotide changes via antigenic shift and drift, resulting in the added diversity and emergence of novel influenza viruses. This further created the possibility of cross-species transmission (3). Of note, avian influenza viruses (AIVs), such as H5N1, H6N1, H7N9, H9N2, and H10N8, can occasionally break the species barrier and spread from domestic poultry to humans, posing a great threat to public health (4–7).

The H7N9 virus is a reassortant AIV consisting of the H9N2 viruses that circulate in the poultry in China and the Eurasian wild bird H7 and N9 viruses (8). In March 2013, the H7N9 virus was first isolated from patients in eastern China (9). Since then, 1,564 patients with H7N9 disease have been reported, and 612 of them were deceased, demonstrating an overall case-fatality ratio of ~38% (10). Sufficient epidemiological data indicate that human infections are directly linked to live-poultry markets (11). Due to several host adaptation mutations and the exchange of internal genes with the H9N2 viruses circulating in the poultry, highly pathogenic AIV (HPAIV) H7N9 viruses emerged during the fifth wave of the H7N9 virus outbreak, characteristically showing a four-amino-acid-insertion at the hemagglutinin (HA) cleavage site (12). During this period, high and low pathogenic H7N9 AIVs co-existed in the poultry.

Studies on highly virulent and pandemic viruses, such as H5N1, have demonstrated specific hallmarks of viral infection, including viral replication rate enhancement and hyper-cytokinemia or “cytokine storm” induction (13, 14). An increasing number of microarray studies performed using animal models have revealed that early and sustained innate immune responses are characteristic of lethal IAV infection (15, 16). Evidence for such responses has been confirmed by counting the lung immune cells via flow cytometry, specifically showing a remarkably increased number of macrophages and neutrophils destined to play critical roles in viral clearance. However, as far as we know, only a cynomolgus macaque model has been used to conduct a study on the gene expression profile changes that occur in H7N9 virus infection (15). Therefore, transcriptome profiles of other animal models are still needed to elucidate H7N9 pathogenesis.

In the present study, we successfully isolated HPAIV H7N9 from the poultry samples collected from Guangxi province. Interestingly, a 12-nucleotide-insertion was observed at the HA cleavage site, and both the HA and neuraminidase (NA) genes were found to belong to the Yangtze River Delta lineage by genomic analysis. This virus not only showed high pathogenicity toward chickens but could also cause lethal infection without prior adaptation in mice. Additionally, it was associated with high pulmonary levels of pro-inflammatory cytokines. Therefore, we aimed to identify host transcriptome responses associated with virological outcomes in IAV H7N9-infected mice. To the best of our knowledge, this study is the first microarray analysis of H7N9-infected mouse lungs, thereby providing a basis for a better understanding of H7N9 pathogenicity in the mouse model.

## Materials and Methods

### Virus

During the fifth wave of the H7N9 outbreak, AIV A/chicken/Guangxi/YL01/2017 (abbreviated as GX/YL01) (accession number EPI_ISL_436706) was isolated from the lungs of dead chickens obtained from Guangxi provinces. The virus was amplified using 10-day-old specific-pathogen-free (SPF) embryonic chicken eggs and then titrated by determining the log_10_[egg infective dose 50% (EID_50_) per milliliter] values. Aliquots of viral stock were stored at −80°C until use. All experiments using the H7N9 virus were performed in a Biosafety Level 3 laboratory (17). This study was performed in accordance with the recommendations by the Biosafety Level 3 laboratory at Huazhong Agricultural University. All procedures were approved by the Institutional Biosafety Committee of Huazhong Agricultural University.

### Genomic Sequencing and Phylogenetic Analysis

Viral RNA was obtained from samples of virus-infected allantoic fluids via the lysis of the viruses using TRIzol LS Reagent (Life Technologies, Inc., CA, USA), as described in a previous study (18). RNA was reverse-transcribed into complementary DNA (cDNA) using 12-base universal primers for IAVs (U12 A/G: AGCG/AAAAGCAGG) and M-MuLV reverse transcriptase (Promega, WI, USA). All segments were amplified using Ex Taq™ DNA polymerase (Takara, Japan) and segment-specific primers (19). The polymerase chain reaction (PCR) products were purified using a Cycle-Pure Kit and a Gel Extraction Kit (Omega Bio-Tek, USA), and the fragments were cloned into a pGEM-T easy vector and sequenced by the dideoxy method using an ABI 3730 DNA sequencer (Applied Biosystems, CA, USA) (19). Data were edited and aligned by BioEdit (version 7.0.5.2).

Phylogenetic analysis was performed based on the open reading frame of the HA and NA genes. Multiple alignments were constructed using the ClustalW multiple alignment tool of BioEdit (version 7.0.5.2). Phylogenetic trees were generated by neighbor-joining bootstrap analysis (1,000 replicates) using the Construct/Test Neighbor-Joining Tree tool of MEGA (version 7.0.14) (20).

### Viral Pathogenicity in Chicken and Mouse Models

In the chicken pathogenicity test, groups of 5 5-week-old SPF white Leghorn chickens were intranasally infected with 10-fold serial dilutions (50 μl) of the H7N9/GX virus (1 ml) at 10^3^-10^6^ EID_50_. The mortality rate was observed for 14 days. Lethal dose 50% (LD_50_) titers were calculated using the method of Reed and Muench, and the LD_50_ value of the virus was expressed as log_10_EID_50_. At 10^6^ EID_50_ infected groups, brain, lungs, spleen, kidneys, and cecum of chicken were collected when they died and stored below −80°C. Then we measured the virus titers in the tissue according to the previous study (12).

In the mouse pathogenicity test, 6-week-old female BALB/c mice (weight, 16–18 g) were intranasally inoculated with 10-fold serial dilutions (50 μl) of the virus at 10^4^-10^8^ EID_50_. Mouse body weight, symptoms, and survival rate were monitored daily for 14 days after virus inoculation. Groups of 6 mice were intranasally inoculated with serial 10-fold dilutions of the virus. Mouse lethal dose 50% (MLD_50_) titers were calculated using the method of Reed and Muench and expressed as the log_10_EID_50_ required to give one unit of MLD_50_ (12).

To compare morbidity (measured by weight loss), mortality, and viral distribution in different tissues, additional mice were inoculated with the virus at 10^6^ EID_50_. Mouse weight loss and mortality rate were monitored daily for 14 days. Mice (*n* = 3 from each group) were killed 1, 3, and 5 days post-infection (dpi), and their blood and lungs were collected. The collected organs were separated into two sets; one set was frozen at −80°C for RNA extraction and other analyses, whereas the other set was fixed in 10% neutral formalin for histopathological study. Viral titers in the tissue homogenates were determined via the EID_50_ titer assay in the SPF chicken eggs (21).

### Determination of the Interferon and Cytokine Levels in the Homogenized Lung Specimens

The pulmonary messenger RNA (mRNA) expression levels of interferon (IFN-β), IFN-γ, interleukin (IL)-1β, IL-6, and tumor necrosis factor (TNF)-α were determined by real-time reverse transcription (RT)-PCR using oligo(dT) transcribed-cDNA obtained from lung tissue homogenates. Glyceraldehyde 3-phosphate dehydrogenase expression was quantified by real-time RT-PCR and used for RNA normalization, and the ΔΔCt method (where Ct is threshold cycle) was used to estimate the differential gene expression between samples (22). Primers for real-time PCR are listed in Table 1.

**Table 1:** Primers used in this study.

**Gene name**	**Sense (5^**′**^-3^**′**^)**	**Anti-sense (5^**′**^-3^**′**^)**
Irf7	CCCCAGCCGGTGATCTTTC	CACAGTGACGGTCCTCGAAG
Cxcl10	ACTGCATCCATATCGATGAC	TTCATCGTGGCAATGATCTC
Usp18	TTGGGCTCCTGAGGAAACC	CGATGTTGTGTAAACCAACCAGA
Herc6	AATTGGTGGCCGTGTTTCAC	CCTGATGAGTTGGGTTGCTTG
Stat1	TCACAGTGGTTCGAGCTTCAG	GCAAACGAGACATCATAGGCA
Stat2	TCCTGCCAATGGACGTTCG	GTCCCACTGGTTCAGTTGGT
Isg15	GGTGTCCGTGACTAACTCCAT	TGGAAAGGGTAAGACCGTCCT
Isg20	TGGGCCTCAAAGGGTGAGT	CGGGTCGGATGTACTTGTCATA
Trafd1	ATGGCCGAGTTTCGAGATGAC	CCTTTGACAGTGGATTTCATGGA
Trim34a	GTAATAACGGTATCTTGGGCTCC	TGCGTTGTCTAACATCAAACCTT
Rsad2	GGTGCCTGAATCTAACCAGAAG	CCACGCCAACATCCAGAATA
Zbp1	GACGACAGCCAAAGAAGTGA	GAGCTATGTCTTGGCCTTCC
Fmo3	GGAAGAGTTGGTGAAGACCG	CCCACATGCTTTGAGAGGAG
Dmpk	CTGCTCGACCTTCTCCTGG	CACGCCCGATCACCTTCAA
Cyp2a5	TGGTCCTGTATTCACCATCTACC	ACTACGCCATAGCCTTTGAAAA
Myh14	CAACCTGCGAGAACGCTACTA	CTTGCCCCGGTACATTTCAAC
Cdc42bpg	TAACGTGGCGCAGTTTCTGAG	CCTCTGCCAATCACCTTCAAGA
TNF-α	CAGGCGGTGCCTATGTCTC	CGATCACCCCGAAGTTCAGTAG
IFN-β	AGCTCCAAGAAAGGACGAACAT	GCCCTGTAGGTGAGGTTGATCT
IL-1β	TGTGAAATGCCACCTTTTGA	GGTCAAAGGTTTGGAAGCAG
IL-6	TCTTGGGACTGATGCTGGTG	TGCCATTGCACAACTCTTTTCT
GAPDH	AGGTCGGTGTGAACGGATTTG	TGTAGACCATGTAGTTGAGGTCA

*GAPDH, Glyceraldehyde 3-phosphate dehydrogenase*.

Protein levels of IFN-γ, IL-1β, IL-6, and TNF-α in the clarified lung homogenates were determined by enzyme-linked immunosorbent assay (ELISA) using Magnetic Luminex® Assay Multiplex Kits (R&D Systems, USA), as described previously (23). Additionally, IFN-β levels in the lung homogenates were determined using a VeriKine Mouse IFN-β ELISA Kit (BioLegend, CA, USA).

### Histopathological Staining

To characterize the pathological features in a mouse infected with the virus at 10^6^ EID_50_, lung tissues were obtained 3 dpi. To examine the pathological changes in the infected mouse lungs 3 dpi, the right side of the lung was fixed in 10% formalin. All four lung lobes were embedded in paraffin and sectioned (5-mm thickness) for hematoxylin and eosin (H&E) staining (23). All lung fields of the four lobes in each sample were examined at 20× magnification.

### RNA Isolation, Library Preparation, and Sequencing

Total RNA was extracted from the samples stored in RNAstore reagent using TRIzol (Invitrogen, USA), according to the manufacturer's protocol. The purity and concentration of the extracted RNA were determined by measuring the optical density at 260 nm (OD260) and 280 nm (OD280) using the NanoDrop 2000 System (Thermo Scientific, MA, USA). RNA integrity was assessed using the RNA Nano 6000 Assay Kit on the Agilent Bioanalyzer 2100 System (Agilent Technologies, CA, USA). RNA (3 μg per sample) was used as the input material, and sequencing libraries were generated using the NEBNext® Ultra™ Directional RNA Library Prep Kit for Illumina® (NEB, MA, USA), following the manufacturer's instructions. High-quality cDNA libraries were then sequenced on an Illumina Hiseq 4000 Sequencer (Illumina, CA, USA), and the base-calling was performed using the software CASAVA (version 1.8.2) (Illumina); as a result, 150-bp paired-end reads were generated (24).

### Transcriptome Data Analysis

Raw data (raw reads) of FASTQ format were first processed using in-house Perl scripts. Clean data (clean reads) were obtained by removing low-quality reads and reads that contained the adapter or ploy-N from the raw data. At the same time, the Q30 value and the GC content of the clean data were calculated. Clean reads were then mapped to the mouse reference genome (GRCm38.p6) downloaded from the National Center for Biotechnology Information (https://www.ncbi.nlm.nih.gov/assembly/GCF_000001635.26) using the software HISAT2 (version 2.0.1). To detect the differentially expressed genes (DEGs), the number of clean reads assigned to a gene was determined using HTSeq (version 0.6.1) and then normalized to the fragments per kilobase of exon per million fragments mapped value. The differential expression levels among the groups were analyzed using the DEseq2 R package (version 1.20.0). DEGs were identified by setting the corrected *P* < 0.05 and |log2 fold change (FC)| >1 as the threshold parameters (25).

Gene Ontology (GO) and Kyoto Encyclopedia of Genes and Genomes (KEGG) analyses of DEGs were performed using the clusterProfiler package, with *P* < 0.05 set as the threshold. GO groups essential for viral immunity with adjusted *P* < 0.05 were visualized using bubble plots. Details of the genes that belonged to particular GO terms and their FC values were presented as circos plots using the “GOplot” library (26).

### Quantitative Real-Time Reverse Transcription-Polymerase Chain Reaction Analysis

RNA was extracted using TRIzol and purified from the final aqueous phase using the RNeasy Mini Kit (Qiagen, Germany). RNA was treated with DNase (Promega), and first-strand cDNA was synthesized using All-in-One cDNA Synthesis SuperMix (Bimake, TX, USA). Relative mRNA expression was determined via SYBR Green-based quantitative RT (qRT)-PCR using SYBR Green SuperMix (Bimake) on an ABI ViiA 7 Real-Time PCR System (Applied Biosystems) (22). Obtained mRNA levels were normalized to the glyceraldehyde 3-phosphate dehydrogenase mRNA level. Gene-specific primers for qRT-PCR are listed in Table 1.

### Statistical Analysis

The results were expressed as the mean ± standard deviation, and all the data were representative of at least three independent experiments. Data analysis was performed using Student's *t*-test. Differences between the mean values were considered significant at *P* < 0.05.

## Results

### Nucleotide Sequencing and Phylogenetic Analysis

The GX/YL01 H7N9 virus was isolated from a dead chicken obtained from the chicken farms in Guangxi province and passaged in SPF embryos. First, the entire genome of the isolated virus was sequenced. Full-genome sequence data for this virus (A/chick/Guangxi/YL01/2017) were submitted to GISAID (https://www.gisaid.org/) and are available under accession number EPI_ISL_436706. According to the BLAST analysis performed for each sequence (https://blast.ncbi.nlm.nih.gov/Blast.cgi), the novel isolated virus belonged to the AIV H7N9 subtype.

**Figure 1:**
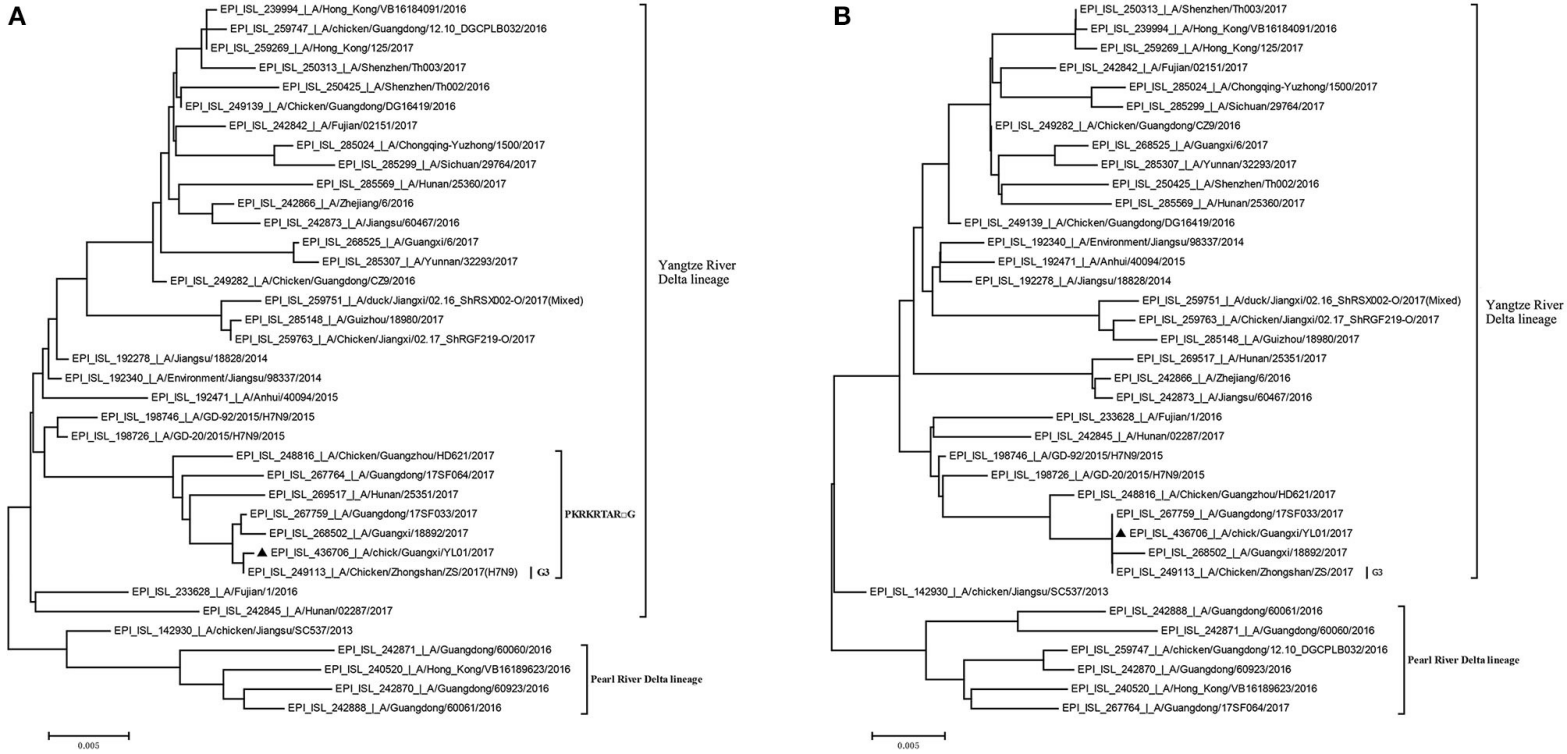
Phylogenies of HA and NA genes of H7N9 viruses from different genotype. All of the H7N9 viruses that represent different genotype viruses prevalent in wave5, including our one new isolate, were used to perform the phylogenetic analysis. Tree was constructed by using the neighbor-joining method with the maximum composite likelihood model in MEGA version 7.0.14 (http://www.megasoftware.net) with 1,000 bp replicates, based on the nucleotide sequence 1–1,695 (HA) and 1–1,398 (NA). Virus lineages are shown at right. Scale bar indicates nucleotide substitutions per site. **(A)** shows the HA gene tree, and **(B)** shows the NA gene tree.

Previously, the genetic analysis indicated that the dynamic reassortment of the internal and surface genes of the Yangtze River Delta and Pearl River Delta lineages between LP-H7N9/H9N2/H6Ny and HP-H7N9 resulted in at least 36 genotypes (12, 27, 28). Using BLAST search in GenBank, we found that six internal genes of the GX/YL01 virus were highly homologous with poultry H9N2 virus (98.46–99.40%) (Supplementary Data 1). To understand the evolutionary relationships in detail, a phylogenetic analysis of the coding regions of the HA and NA genes was conducted. As shown in Figure 1, both HA and NA of the GX/YL01 H7N9 isolate belonged to the Yangtze River Delta lineage.

Similar to the previously isolated HPAIV H7N9, GX/YL01 possessed an HA gene that belonged to the G3 genotype (the represented strain: A/Chicken/Zhongshan/ZS/2017-like) and a four-amino-acid-insertion at the cleavage sites (Pkr*krta*rg) (Table 2). This insertion is characteristic of highly pathogenic avian species (12, 29). Unlike the A/Anhui/40094/2015 (GISAID ID: EPI_ISL_192471) HA protein, which showed an amino acid mutation (Q226L) at the HA receptor-binding site, increasing its binding affinity to 2,6-linked sialic acids (characteristic of human cell-surface receptors), the GX/YL01 HA gene showed a Q226 amino acid mutation (30).

**Table 2:** Molecular characteristics of the H7N9 viruses isolated from poultry.

**Genes and sites**		**Function**	**H7N9 virus**		
			**A/chick/Guangxi/YL01/2017**	**A/Chicken/Zhongshan/ZS/2017^**a**^**	**A/Anhui/40094/2015^**b**^**
HA	**Cleavage site**		**PKRKRTARG**	**PKRKRTARG**	**PKG—RG**
	Residues 224-228^c^	Favor mammalian adaptation, Receptor binding site	NG**Q**SG	NG**Q**SG	NG**L**SG
NA	R292K^d^	Related to drug resistance	R	R	R
	Stalk deletion		69–73	69–73	69–73
M2	S31N	Reduced sensitivity to amantadine	N	N	N
PB2	K526R	Enhance the 627K and 701N function	R	R	K
	A588V	Promotes the mammalian adaptation and virulence in mice	A	A	V
	E627K	Increased virulence in mice	E	E	K
	D701N	Enhanced transmission in guinea pigs	D	D	D

a *Regard as a high pathogen to chicken, belong to G3 genotype*;

b *a human origin virus and is a low pathogen to chicken, belong to G11 genotype*;

c *In H7, numbering is residues 233–237*;

d *In H7, numbering is residues 289*.

Similarly, the isolated GX/YL01 H7N9 virus possessed the amantadine resistance mutation (S31N) in M2 but not the NA inhibitor resistance mutation (R292K). Additionally, a five-amino-acid-deletion at positions 69–73, often observed in other H7N9 viruses, was observed in the NA stalk of GX/YL01. It has been documented that all the emerging human isolates possessed at least one of the four mammalian adaptation mutations, i.e., K526R, A588V, E627K, or D701N, in PB2. Nevertheless, the GX/YL01 H7N9 virus of avian origin possessed only one mutation (K526R) (Table 2).

### H7N9 Viral Replication and Virulence in Chickens

**Figure 2:**
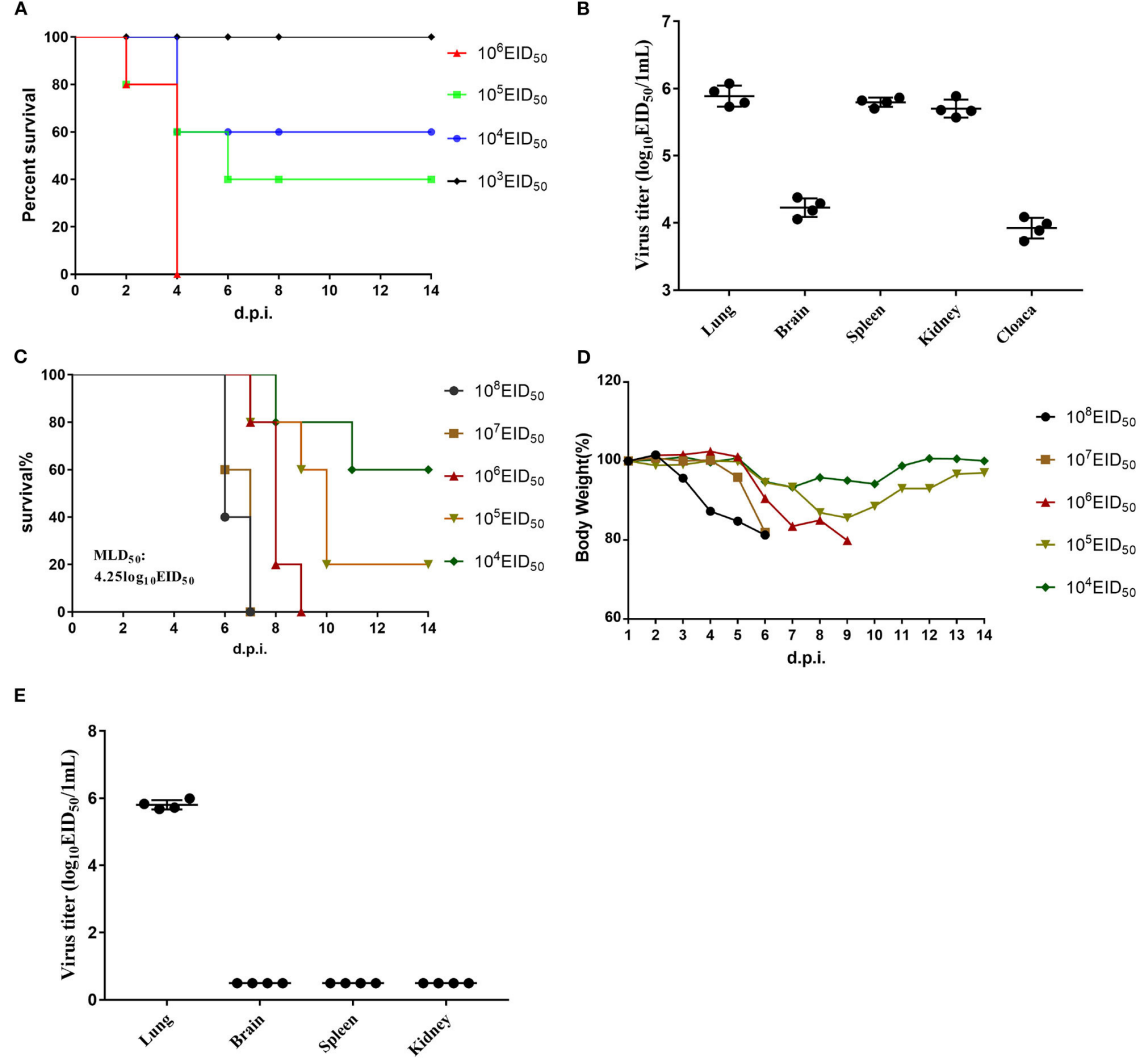
Challenge of chickens and mice with isolated H7N9 viruses. **(A)** Mortality of inoculated chickens. Groups of five 5-week-old SPF white leg-horn chickens were inoculated intranasally with 50 μl 10-fold serial dilutions containing 10^3^-10^6^EID_50_ of H7N9/GX. **(B)** Virus titers in the lungs, brains, spleen, kidney, and cloaca of the first four dead chickens in 10^6^EID_50_ of H7N9/GX infected group. **(C)** Mortality of inoculated mouse. Groups of eight 6-week-old SPF BALB/c mice were inoculated intranasally with 50 μl 10-fold serial dilutions containing 10^3^-10^6^EID_50_ of H7N9/GX. **(D)** Weight changes in the mice. **(E)** Virus titers in the lungs, brains, spleen, and kidney of the first four dead mice in 10^6^EID_50_ of H7N9/GX-infected group.

The EID_50_ value of GX/YL01 was 8.17, which was calculated using the method of Reed and Muench and expressed as log10. To determine GX/YL01 viral replication and pathogenicity in chickens, groups of 5-week-old SPF white leg-horn chickens were intranasally inoculated with 10-fold serial dilutions (50 μl) of the GX/YL01 virus at 10^3^-10^6^ EID_50_. As shown in Figure 2A, the LD_50_ value in SPF chicken was 4.5, and 100% of the chickens infected with the virus at 10^6^ EID_50_ succumbed by 4 dpi.

We collected the brain, lungs, spleen, kidneys, and cecum of each chicken inoculated with the virus at 10^6^ EID_50_ and determined the viral titers in the eggs. Mean viral titers ranging from 3.7 to 6.0 log_10_EID_50_/g were detected in all the organs mentioned earlier (Figure 2B). Therefore, we concluded that GX/YL01 could systemically infect chickens after intranasal viral inoculation, thereby indicating that the GX/YL01 virus was highly pathogenic toward chickens.

### Analysis of Viral Pathogenicity in Mice

The multiple basic amino acid motifs in the HA cleavage site of AIV H7N9 is a prerequisite for its lethality in mammals (12, 31, 32). To evaluate whether the four-amino-acid-insertion at the HA cleavage site increased GX/YL01 virulence in mammals, we tested viral replication and virulence in mice. Weight loss of infected mouse was monitored daily for 14 dpi, and MLD_50_ values were determined (Figure 2C). Consistent with previous studies, HPAIV GX/YL01 inoculation caused significant weight loss, and the MLD_50_ value was calculated to be 4.25 log_10_EID_50_/ml (Figure 2D). We collected the brain, lungs, spleen, and kidneys of each mouse inoculated with the virus at 10^6^ EID_50_ and determined the viral titers in the eggs. As expected, high levels of infectious GX/YL01 were successfully rescued from the lung samples but not the spleen, kidney, or brain samples (Figure 2E).

**Figure 3:**
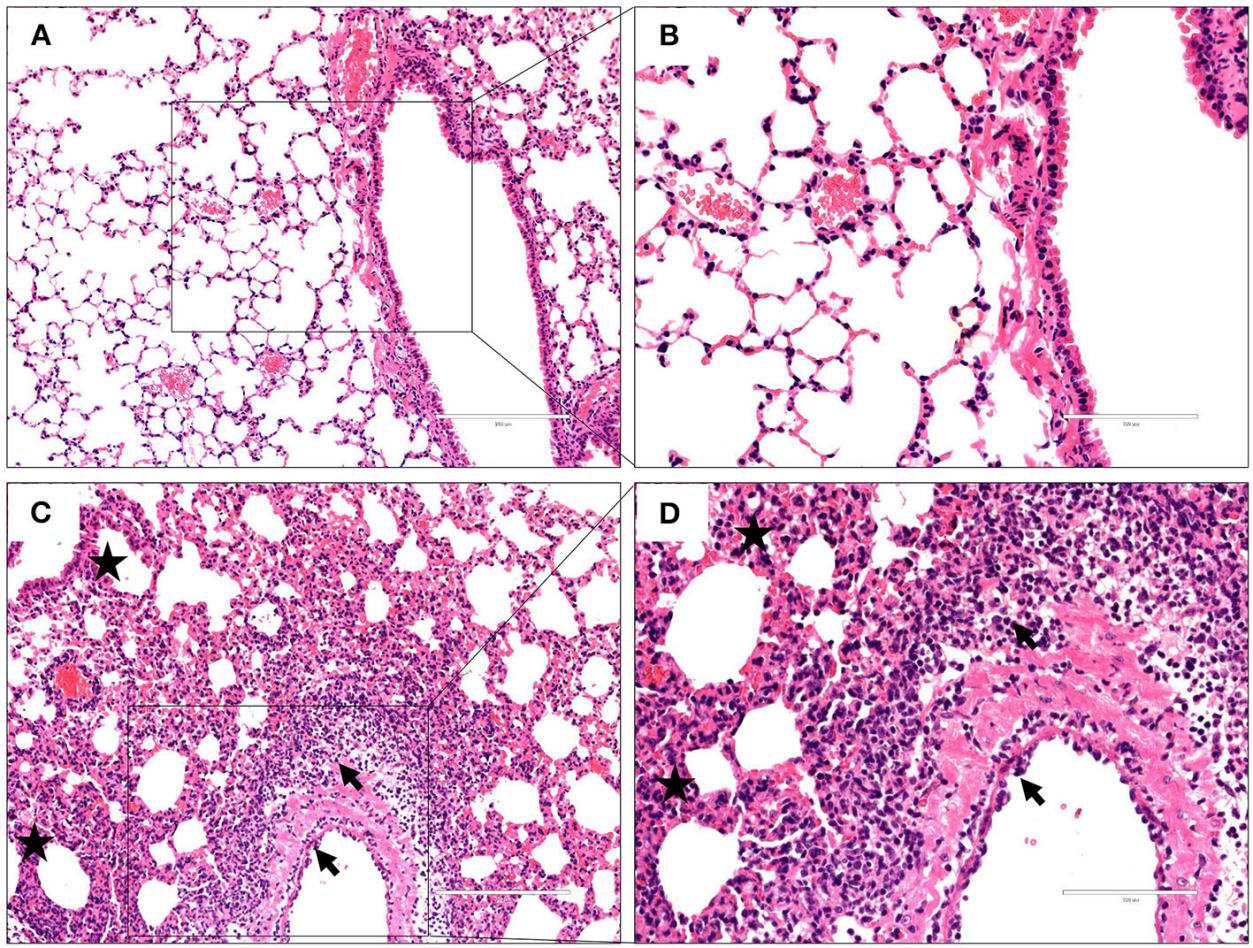
Histopathological changes in hematoxylin and eosin-stained lung tissues. Representative histopathological changes in hematoxylin and eosin-stained lung tissues from a mouse injected with PBS **(A,B)** or 10^6^EID_50_ of H7N9/GX virus **(C,D)** 3 days postinfection. Pulmonary alveolar hemorrhage, bronchial mucosa injury, and accompanied by inflammatory cells infiltration around the bronchus (shown as a black arrow), obviously interstitial hyperplasia is captured, and accompany with lymphocytes and dust cells infiltration (shown as asterisk) **(C,D)**. No obvious histopathology was observed in the PBS-incubated mouse lung **(A,B)**.

To characterize the pathological features in mice infected with the virus at 10^6^ EID_50_, lung tissues were obtained 3 dpi and subjected to histopathological analysis via H&E staining. Histopathological changes in mouse lungs were similar to those exhibited in human lungs; the GX/YL01 virus induced severe lung injury, represented by pulmonary alveolar hemorrhage, bronchial mucosa injury with accompanying inflammatory cell infiltration around the bronchus, and obvious interstitial hyperplasia with accompanying lymphocyte and dust cell infiltration (Figures 3C,D). On the contrary, no obvious histopathology was observed in the PBS-incubated mouse lungs (Figures 3A,B). Altogether, these results demonstrated that the GX/YL01 virus was virulent in mice, showing high lung viral titers and severe lung injury.

### Identification and Enrichment Analysis of Differentially Expressed Genes

**Figure 4:**
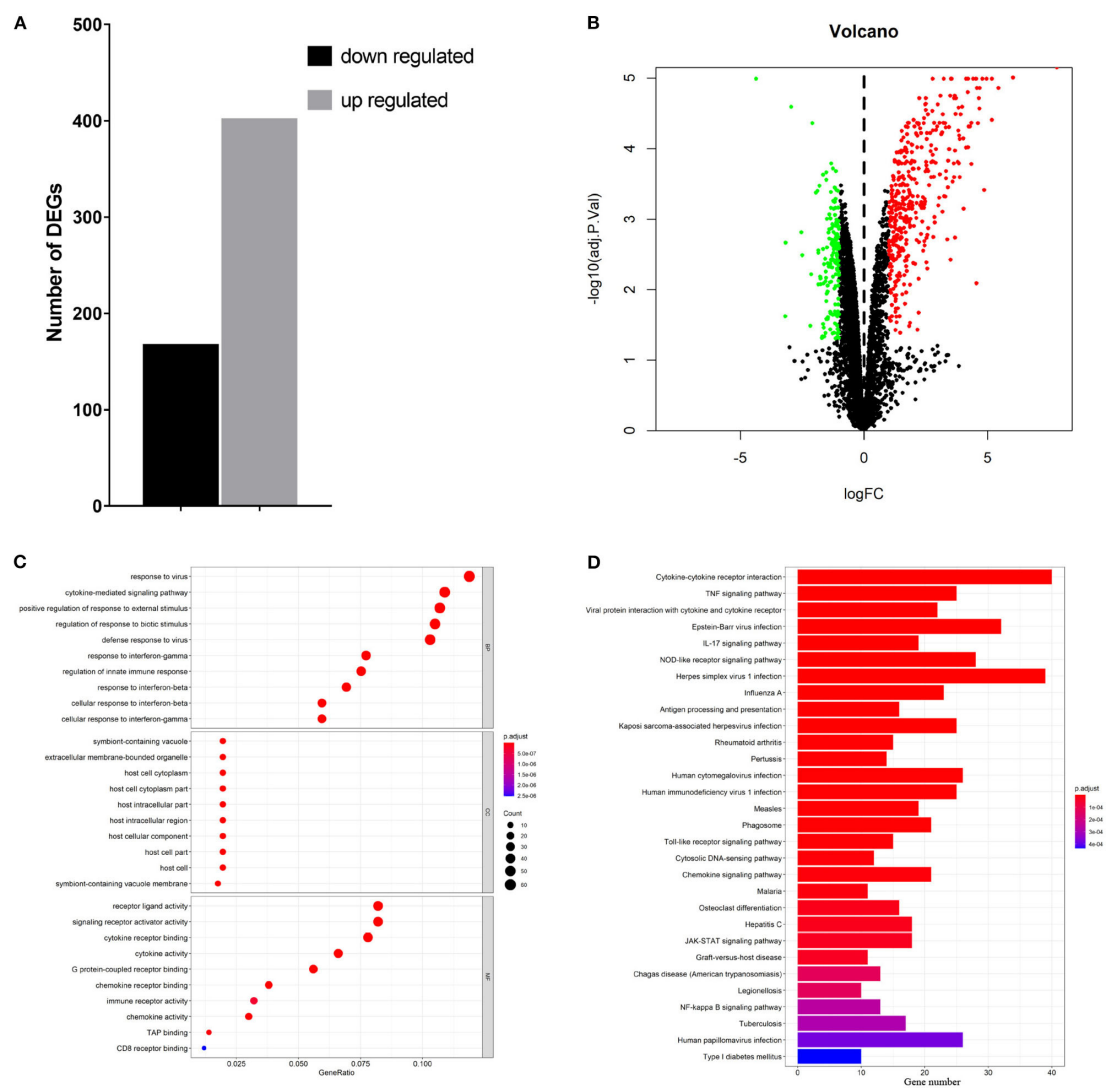
Transcriptome data profile generated by Illumina sequencing and differential expression analysis. **(A)** Numbers of differentially expressed genes. **(B)** Volcano map showing the significant differentially expressed genes between PBS or 10^6^EID_50_ of H7N9/GX virus-infected mouse lung tissue. Red and green dots represent upregulated and downregulated genes, respectively, whereas the black dots represent microRNAs with no significant difference (*P* < 0.05 and |log2FC| ≥ 1 as the threshold cutoff). **(C)** Gene Ontology functional annotation and classification of differentially expressed genes. Functional classification of DEGs was performed using GO analysis (http://www.geneontology.org/) and the categories “biological process (BP),” “molecular function (MF),” and “cellular component (CC)” were analyzed. X-axis is the enrichment ratio (gene ratio); calculated as the ratio of the number of genes annotated to an entry in the selected gene set to the total number of genes annotated to the entry in the species. Size of the bubble represents the number of genes annotated to GO terms. Color represents the enrichment Q value. **(D)** Kyoto Encyclopedia of Genes and Genomes (https://www.kegg.jp/) functional annotations of the DEGs (top 30). A bar chart shows enrichment of the DEGs in the signaling pathways, the horizontal axis shows the number of genes, and the vertical axis shows each pathway. Color represents the enrichment value.

To elucidate global host responses specifically associated with virus-induced lung injury sites in GX/YL01-infected mice, we used microarrays to assess the transcriptional profiles of the lung lesions. Mice (*n* = 3 from each group) were killed 3 dpi, and lungs were collected to determine the transcriptional cytokine response. Six cDNA libraries prepared using GX/YL01 virus-infected (GX_72) and non-infected (CK_72) mouse lungs were sequenced and filtered, yielding ~87–108 million clean reads per sample (Table 3). The transcripts were filtered by setting *P* ≤ 0.05 and |log2 FC| ≥ 1 as the thresholds. In total, 54,298 transcripts were identified via RNA sequencing analysis, and 566 of them were identified as DEGs between the virus-infected and non-infected mice (Supplementary Data 2). Among these, 400 genes were upregulated, showing log2 FC values ranging from 1 to 7.8, whereas the remaining 166 genes were downregulated, showing log2 FC values ranging from −4.3 to −1, as shown in the volcano map (Figures 4A,B). Meanwhile, the expression levels of 17 representative DEGs were further verified by qRT-PCR, confirming the accuracy and reliability of the transcriptome data (Table 4).

**Table 3:** Statistics of the RNA-seq datasets.

**Sample**	**Raw_reads**	**Clean_reads**	**Total mapped**	**Error_rate(%)**	**Q30(%)**	**GC_content(%)**
CK_72_1	94920470	91327754	84112355 (92.1%)	0.01	94.2	49.39
CK_72_2	90704838	87021076	81028155 (93.11%)	0.01	94.13	47.94
CK_72_3	108007470	103763534	96295286 (92.8%)	0.01	94.06	48.47
GX_72_1	110626548	108676024	99764250 (91.8%)	0.01	93.38	48.87
GX_72_2	102186552	97821506	90036280 (92.04%)	0.01	93.91	48.6
GX_72_3	96141762	91906468	84107898 (91.51%)	0.01	94.31	49.31

**Table 4:** Validation of DEGs in RNA-seq by qRT-PCR.

**Gene name**	**RNA-seq**		**qRT-PCR**
	**Log_**2**_FC**	**adj.P.Val**	**Fold ± SD**
Irf7	4.54	1.02E-05	53.04 ± 3.92
Cxcl10	6.04	9.90E-06	135.47 ± 3.77
Usp18	3.73	1.93E-05	12.62 ± 1.4
Herc6	2.77	2.92E-05	8.23 ± 0.91
Stat1	2.55	1.97E-04	8.51 ± 0.15
Stat2	2.48	2.38E-05	5.42 ± 0.17
Isg15	5.18	1.02E-05	72.59 ± 5.34
Isg20	1.91	2.61E-04	10.81 ± 1.24
Trafd1	1.81	6.15E-04	2.43 ± 0.11
Trim34a	1.18	2.45E-04	6.53 ± 0.58
Rsad2	4.69	1.39E-05	14.50 ± 1.30
Zbp1	4.68	2.72E-05	22.29 ± 0.86
Fmo3	−2.53	1.54E-03	−4.34 ± 0.20
Dmpk	−2.94	2.55E-05	−4.64 ± 0.53
Cyp2a5	−3.17	2.17E-03	−7.61 ± 0.8
Myh14	−3.19	2.39E-02	−7.92 ± 0.73
Cdc42bpg	−4.36	1.02E-05	−7.74 ± 0.69

To better understand the host's response to the GX/YL01 virus infection, GO analysis was conducted (Figure 4C). The genes that were differentially expressed during this experiment are involved in biological processes, such as “response to virus,” “cytokine-mediated signaling pathway,” “positive regulation of cytokine production,” “regulation of response to biotic stimulus,” “defense response to virus,” and “response to interferon-beta” (Supplementary Data 3). Then, to identify the biological pathways in which the DEGs participated, KEGG pathway/enrichment analysis was performed. As shown in Figure 4D, DEGs were primarily involved in either host immune response or disease pathways, including “cytokine–cytokine receptor interaction,” “TNF signaling pathway,” “viral protein interaction with cytokine and cytokine receptor,” “Epstein–Barr virus infection,” “herpes simplex virus 1 infection,” and “influenza A” (Supplementary Data 4).

### Interferon-Stimulating Genes (ISGs) and Cytokines Associated With Multi-Gene Ontology Terms Were Significantly Upregulated

**Figure 5:**
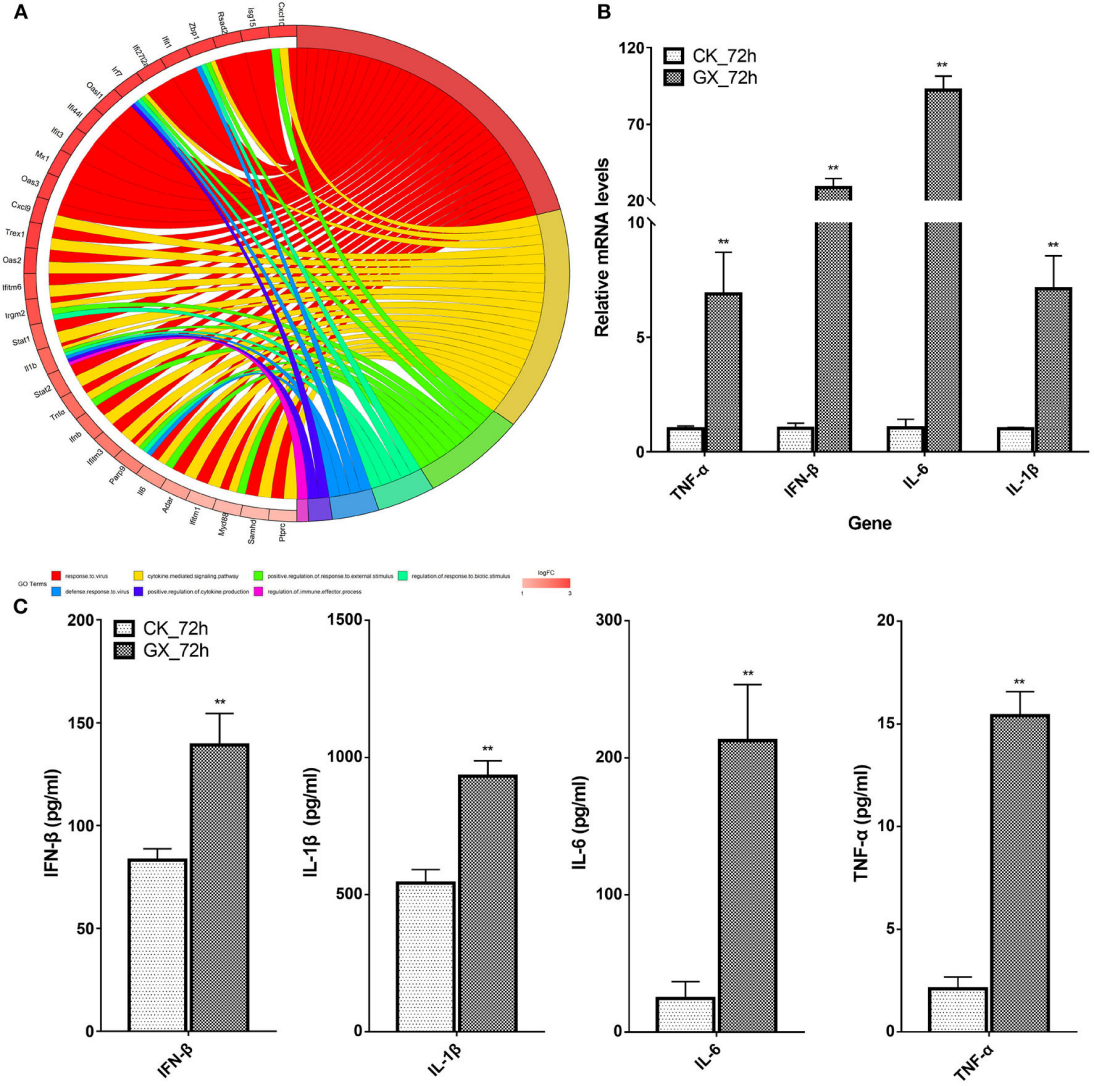
Most frequent genes in the Top7 Gene Ontology terms. **(A)** Circos plots of virus immunity-related Gene Ontology terms. Line color represents different Gene Ontology terms listed in legend; box color grid indicates the gene expression levels. **(B)** Relative messenger RNA levels of interferon-β, interleukin (IL)-1β, interleukin-6, and tumor necrosis factor-α genes in mouse lung were determined by real-time reverse transcription-polymerase chain reaction. Mouse glyceraldehyde 3-phosphate dehydrogenase messenger RNA was used for RNA concentration normalization. Error bar indicates ± SD. (means ± SD from three independent experiments) (*P < 0.05; **P < 0.01; all by two-tailed Student's t test). **(C)** Protein levels of cytokines interferon-β, IL-1β, IL-6, and tumor necrosis factor-α presented in mouse lung homogenates were determined by enzyme-linked immunosorbent assay. Lungs from infected mice (3–5 mice from each group) were homogenized in 1 ml of phosphate-buffered saline. Clarified homogenates were used for cytokine detection.

Based on the earlier mentioned KEGG analysis results, we found that the DEGs were mainly related to immunity and antivirus pathways; thus, GO plot analysis was performed to present further details of the genes, such as the most popular genes related to the top GO terms and their gene expression levels. As shown in Figure 5A, genes associated with at least two GO terms were listed. These genes included IFN-stimulating genes (ISGs), z-DNA-binding protein 1, C-X-C motif chemokine 10, IFN regulatory factor 7, myeloid differentiation primary response gene 88, and IFN-induced transmembrane protein 3. On the other hand, cytokines, including IL-6, IL-1β, IFN-β, and TNF-α, were upregulated in response to H7N9 infection. Finally, the mRNA and protein levels of the selected cytokines in the GX/YL01 virus-infected lungs were tested by qPCR and ELISA, respectively. Significantly increased levels were exhibited in response to viral infection (Figures 5B,C).

## Discussion

Since its first emergence in China in 2013, the novel AIV of the H7N9 subtype has been of great concern worldwide. The human H7N9 virus is a reassortant virus with the surface HA and NA genes derived from duck and migratory bird viruses, respectively, and six internal genes originating from two different groups of H9N2 viruses circulating in the poultry in China. In this study, we isolated a highly pathogenic pure avian H7N9 virus (GX/YL01), which could replicate effectively in the mouse lung, causing severe pneumonia. Additionally, host innate immune response dysregulation and cytokine induction in mice were found to be similar to that in humans. Importantly, we found that over-activated antiviral signals and intense ISG products possibly contributed to the high virulence of the virus in mice.

Initially, H7N9 viruses were non-pathogenic in chickens; however, they evolved into highly pathogenic viruses, causing severe outbreaks in chickens during the fifth wave of the H7N9 virus outbreak. Indeed, the virus isolated in this study was highly pathogenic in SPF chickens (Figures 2A,B). The increased viral virulence resulted from several adaptive mutations. First, a four-amino-acid-insertion at the HA cleavage site, regarded as characteristic of highly pathogenic avian viruses, was observed in the GX/YL01 HA. Additionally, other adaptive mutations in the gene fragments contributed to the high pathogenicity of avian influenza strains. For example, the HA receptor-binding site mutation (Q226L), NA stalk deletion, and PB2 mutations (K526R, A588V, E627K, and D701N) promoted mammalian adaptation and increased viral virulence in mice (33). Unlike the L226 mutation at the receptor-binding site in the low pathogenic avian H7N9 virus, the Q226 mutation in the GX/YL01 HA resulted in a high binding affinity toward 2,3-linked sialic acids; this is characteristic of avian cell surface receptors. Taken together, the insertion at the HA cleavage site and the high binding affinity of avian cell receptors might explain the high pathogenicity of the virus in poultry.

Patients infected with H7N9 viruses often display a severe and lethal disease characterized by rapidly progressive pneumonia that results in acute respiratory distress syndrome and respiratory failure (34). To check whether the virus-infected mice developed pneumonia, we detected the histopathological changes in the H&E-stained lung tissues of the mice intranasally infected with the GX/YL01 virus at 10^6^ EID_50_. As expected, diffuse alveolar damage, pulmonary alveolar hemorrhage, bronchial mucosa injury, inflammatory cell infiltration around the bronchus, and obvious interstitial hyperplasia could be observed in the virus-infected mouse lung tissues (Figure 3). However, clinically and histopathologically, H7N9 infection was not as severe in mice as it is in humans. Although viral characteristics could most likely be responsible for the differences in disease severity between mice and humans, underlying medical complications in humans might be another reason.

Several studies have demonstrated that high viral replication, lymphopenia, “cytokine storm” induction, macrophage replication, and viral dissemination beyond the respiratory tract are associated with increased severity of the H5 and H7 disease in humans and animal models (29, 35). Investigation of these features in pure HPAIV H7N9 infection warrants further study. To detail the host response to the infection, a transcriptome analysis of the virus-infected or control mouse lung was performed. Enrichment analysis of DEGs showed that some antiviral biological pathways, including “response to virus,” “cytokine-mediated signaling pathway,” “positive regulation of cytokine production,” “regulation of response to biotic stimulus,” “defense response to virus,” and “response to IFN-β” were enriched. GO plot analysis results showed that some genes, which we call “hot genes,” participated in multiple pathways. These genes could be divided into two categories, ISGs and cytokines, according to their functions. It has been shown that cytokine dysregulation is a contributory factor to H5N1- and H7N9-related disease pathogenesis. Our results also showed that pure HPAIV H7N9 infection induced high pulmonary levels of cytokines, including IL-6, IL-1β, IFN-β, and TNF-α. These results are consistent with the published report (15).

In conclusion, the present study reported the mRNA and cytokine expression profiles of pure HPAIV H7N9-infected mouse models for the first time. The results suggested that host innate immune response dysregulation and cytokine induction might play key roles in enhancing viral virulence in mice. This information will be critical for advancing our understanding of the pathogenicity of avian-origin H7N9 viruses in mammals.

## Data Availability Statement

The datasets generated for this study can be found in NCBI SRA, NCBI Accession No. PRJNA662664.

## Ethics Statement

The animal study was reviewed and approved by All animal experiments were approved by the Research Ethics Committee, Huazhong Agricultural University, Hubei, China (HZAUMO-2016-022) and were performed in accordance with the Guidelines for the Care and Use of Laboratory Animals of the Research Ethics Committee, Huazhong Agricultural University, Hubei, China.

## Author Contributions

WG, HK, and ZZ: conceptualization and methodology. YZ, CL, and KH: software. WG and ZZ: formal analysis and writing—original draft preparation. WG, ZZ, and HM: investigation. ZZ, MJ, and YW: writing—review and editing. XYZ and MJ: supervision. WG, ZZ, MJ, and YW: project administration. ZZ and MJ: funding acquisition. All authors: have read and agreed to the published version of the manuscript.

## Conflict of Interest

The authors declare that the research was conducted in the absence of any commercial or financial relationships that could be construed as a potential conflict of interest.
