# Supplementary material for: Transcriptome Profiles of Highly Pathogenic Pure Avian H7N9 Virus-Infected Lungs of BALB/c Mice
Source: Front Vet Sci. 2020 Dec 21;7:603584. doi: 10.3389/fvets.2020.603584 (PMC7779551; doi:10.3389/fvets.2020.603584)
Supplement: Supplementary file 1 [file Table_1.DOCX]

Supplement data 1 The highest nucleotide sequence identity to the new isolated H7N9 AIV was determined by using BLAST search in GenBank. (including H7N9 subtype and none-H7N9 subtype viruses).

| **Gene** | **Closest viruses in GeneBank** | | |
| --- | --- | --- | --- |
|  | **Strain** | **Accession ID** | **Nucleotide identity%** |
| HA | A/chicken/Guangxi/97/2017(H7N9) | MK453329.1 | 100.00% |
|  | A/duck/Fujian/SE0195/2018(H7N2) | MH209523.1 | 99.05% |
| NA | A/chicken/Guangdong/S11523/2017(H7N9) | MH209293.1 | 100.00% |
| PB2 | A/chicken/Guangxi/GX110/2017(H7N9) | MG575547.1 | 99.69% |
|  | A/chicken/Zhejiang/SIC40/2015(H9N2) | KX598552.1 | 98.90% |
| PB1 | A/chicken/Guangxi/GX110/2017(H7N9) | MG575558.1 | 98.86% |
|  | A/chicken/China/G1773PB1,16GD/2016(H9N2) | MK326882.1 | 98.46% |
| PA | A/chicken/Guangxi/GX102/2017(H7N9) | MG575571.1 | 99.77% |
|  | A/chicken/China/F1303/2015(H9N2) | MN100409.1 | 99.12% |
| NP | A/chicken/Guangxi/GX110/2017(H7N9) | MG575591.1 | 99.93% |
|  | A/chicken/Zhejiang/925134/2014(H9N2) | KU042599.1 | 99.06% |
| M | A/chicken/Guangxi/GX110/2017(H7N9) | MG827382.1 | 99.80% |
|  | A/chicken/China/355/2017(H9N2) | MN385382.1 | 99.08% |
| NS | A/chicken/Guangxi/GX110/2017(H7N9) | MG575624.1 | 99.76% |
|  | A/chicken/Zhejiang/727198/2014(H9N2) | KU042648.1 | 99.40% |
